# Gut microbiomes of sympatric Amazonian wood‐eating catfishes (Loricariidae) reflect host identity and little role in wood digestion

**DOI:** 10.1002/ece3.6413

**Published:** 2020-05-25

**Authors:** Mark McCauley, Donovan P. German, Nathan K. Lujan, Colin R. Jackson

**Affiliations:** ^1^ Department of Biology University of Mississippi University MS USA; ^2^ Department of Ecology and Evolutionary Biology University of California Irvine CA USA; ^3^ Department of Ichthyology American Museum of Natural History New York NY USA

**Keywords:** Amazon basin, detritivory, microbiome, Neotropics, phylosymbiosis, wood‐eating catfish

## Abstract

Neotropical wood‐eating catfishes (family Loricariidae) can occur in diverse assemblages with multiple genera and species feeding on the same woody detritus. As such, they present an intriguing system in which to examine the influence of host species identity on the vertebrate gut microbiome as well as to determine the potential role of gut bacteria in wood digestion. We characterized the gut microbiome of two co‐occurring catfish genera and four species: *Panaqolus albomaculatus*, *Panaqolus gnomus*, *Panaqolus nocturnus,* and *Panaque bathyphilus*, as well as that of submerged wood on which they feed. The gut bacterial community did not significantly vary across three gut regions (proximal, mid, distal) for any catfish species, although interspecific variation in the gut microbiome was significant, with magnitude of interspecific difference generally reflecting host phylogenetic proximity. Further, the gut microbiome of each species was significantly different to that present on the submerged wood. Inferring the genomic potential of the gut microbiome revealed that the majority of wood digesting pathways were at best equivalent to and more often depleted or nonexistent within the catfish gut compared to the submerged wood, suggesting a minimal role for the gut microbiome in wood digestion. Rather, these fishes are more likely reliant on fiber degradation performed by microbes in the environment, with their gut microbiome determined more by host identity and phylogenetic history.

## Graphical abstract

We describe the gut microbiomes of four co‐existing freshwater catfish species as well as that of the submerged wood on which they feed. Bacterial communities did not vary by gut region for any catfish species, although they were all different to those identified on submerged wood. Wood digesting pathways were functionally predicted for the wood bacterial microbiome, but were less predicted for the catfish gut microbiome supporting the hypothesis that wood‐eating catfishes do not rely on their gut microbiota to digest wood.

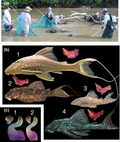

## 1 INTRODUCTION

Vertebrate digestive tracts contain a diverse community of microorganisms that forms the gut microbiome. While microbiome research on nonhuman vertebrates has increased in recent years, an ongoing question is the influence of diet or environmental factors on the gut microbiome in comparison to host identity or evolutionary history (i.e., phylogeny; Colston & Jackson, 2016). The role of host phylogeny is complicated as it may reflect vertical transmission from parent to offspring, coevolution between hosts and their microbiome, or ecological filtering for gut bacteria based on environmental conditions within the host (Mazel et al., 2018; Moran, Ochman, & Hammer, 2019). Regardless, the concept of phylosymbiosis, that there can be an eco‐evolutionary pattern between host phylogenetic relationships and their microbiomes, has gradually emerged (Brooks, Kohl, Brucker, van Opstal, & Bordenstein, 2016; Brucker & Bordenstein, 2012; Kohl, Dearing, & Bordenstein, 2018; Sanders et al., 2014). However, phylogenetic relationships in microbiome composition have not always been observed (Chandler, Lang, Bhatnagar, Eisen, & Kopp, 2011; Dietrich, Koehler, & Brune, 2014), and examples abound of gut microbial communities matching more with geography than host species (Godoy‐Vitorino, Leal, et al., 2012; Hird, Carstens, Cardiff, Dittmann, & Brumfield, 2014), or with combinations of geography, diet, and the animal's evolutionary history all playing roles (Antonopoulou et al., 2019; Belkova et al., 2017; Godoy‐Vitorino, Goldfarb, et al., 2012; Kohl, Varner, Wilkening, & Dearing, 2018).

**Figure 1:**
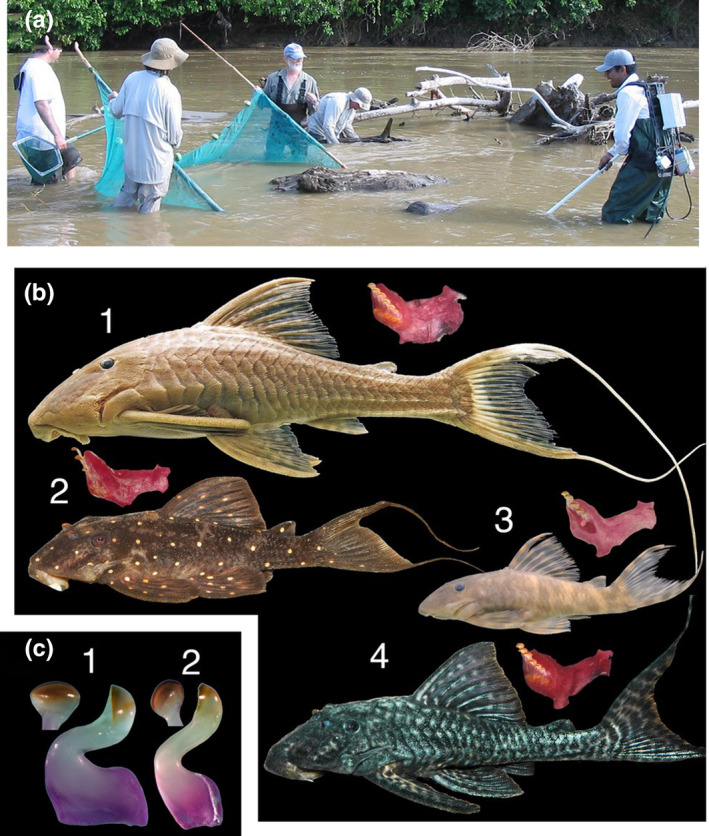
(a) Coarse woody debris habitat in the middle Marañon River basin of northern Peru where wood‐eating catfish specimens were collected for this study. (b) Four species of wood‐eating catfishes examined in this study shown with dissected right mandibles in ventral view: (1) *Panaque bathyphilus*, (2) *Panaqolus albomaculatus*, (3) *Panaqolus gnomus*, and (4) *Panaqolus nocturnus*. (C) Whole teeth of wood‐eating catfishes in lateral view and cusps in occlusal view: (1) Tooth of *Panaque bathyphilus*, (2) tooth of *Panaqolus albomaculatus*

Habitats with shared resources and closely related species offer the chance to test many of the hypotheses surrounding gut microbiome communities. For instance, neotropical wood‐eating catfishes (family Loricariidae, subfamily Hypostominae; Lujan, Cramer, Covain, Fisch‐Muller, & López‐Fernández, 2017) represent an intriguing opportunity to examine the effects of phylogeny versus. diet on gut microbiomes, as paraphyletic assemblages of these catfishes co‐occur and feed on the same unusual food resource: woody detritus (Lujan, German, & Winemiller, 2011). Wood‐eating catfishes comprise three genera (*Cochliodon*, *Panaqolus*, and *Panaque*) and ~24 described species (Lujan et al., 2017) that are among the most distinctive organisms in the Amazon, the Earth's most biologically diverse freshwater ecosystem. All have teeth shaped like a carpentry adz (Figure 1), jaw bones and muscles specialized for gouging surfaces of submerged tree trunks (Lujan & Armbruster, 2012), and approximately 70% of their food intake is composed of wood particles with the remainder composed of amorphous detritus and diatoms (German, 2009; Schaefer & Stewart, 1993). In various parts of the upper Amazon basin, where submerged wood is abundant and represents a major carbon reservoir (Wohl, Dwire, Sutfin, Polvi, & Bazan, 2012), multiple species representing all three genera can be observed feeding on the same log.

Despite their diet, studies have shown little digestion and assimilation of wood by wild‐caught and captive wood‐eating catfishes (German, 2009; German & Bittong, 2009; German & Miles, 2010; Lujan et al., 2011), which suggests little role of enteric microorganisms in the digestion of wood. However, work on the gut microbiome of these animals has been limited to specimens obtained via the aquarium trade (Di Maiuta, Schwarzentruber, Schenker, & Schoelkopf, 2013; McDonald, Schreier, & Watts, 2012; Nelson, Wubah, Whitmer, Johnson, & Stewart, 1999; Watts, McDonald, Daniel, & Schreier, 2013). Given captive husbandry can change an organism's microbiome (Clements, Angert, Montgomery, & Choat, 2014; Fishelson, Montgomery, & Myrberg, 1985; Montgomery & Pollak, 1988), it is imperative to investigate the microbiomes of wild‐caught wood‐eating catfishes for comparison.

Here, we examined whether diet or host identity plays a stronger role in affecting the gut microbiomes of wild‐caught individuals of four sympatric species of wood‐eating catfishes representing two of the three independent evolutionary events leading to the wood‐eating phenotype (Figure 1). We hypothesized that geographic and dietary similarities among the species would lead to them having similar gut microbiomes. We also examined the microbiome in the proximal, mid, and distal regions of the intestines and compared these to each other and to the bacterial community of the wood surfaces on which these fishes were feeding when collected. Based on current literature on these animals (Di Maiuta et al., 2013; McDonald et al., 2012; Nelson et al., 1999; Watts et al., 2013), we hypothesized that they curate a specific bacterial microbiome that is different from that on the wood on which they feed. Nonruminant herbivores typically rely on microbial symbionts in their hindguts to aid in the digestion of plant material, and their hindgut microbiome contains metabolic pathways for the digestion and metabolism of plant fibers (Karasov & Martínez del Rio, 2007; Ley, Lozupone, Hamady, Knight, & Gordon, 2008; Moran, Turner, & Clements, 2005; Moran et al., 2019; Stevens & Hume, 1998). However, because previous studies have shown a lack of wood digestion in wood‐eating catfish guts (German, 2009; German & Bittong, 2009; German & Miles, 2010), we hypothesized that wood‐degrading capabilities would not be enriched in the fishes’ hindgut microbial communities and that microbial communities would differ little among gut regions. Beyond the novel geographic, dietary, and taxonomic aspects of this investigation, this study is one of few to examine the microbiome of herbivorous/detritivorous organisms that appear to rely little on their microbiome for the digestion of plant material, a topic of broad ecological and evolutionary relevance (Moran et al., 2019).

## 2 MATERIALS AND METHODS

### 2.1 Sampling

Individuals of the wood‐eating catfish lineages *Panaqolus* (*Pqs. albomaculatus*, *Pqs. gnomus*, *Pqs. nocturnus*) and *Panaque* (*Pqe*. *bathyphilus*; *n* = 3 individuals per species) were collected in August 2006 (dry season) by electrofishing among partially submerged dead logs and branches in middle reaches of the Marañon River, a tributary of the upper Amazon River, in Condorcanqui Province, Amazonas Department, northern Peru. Specimens were collected from a single coarse woody debris dam at approximately 4°35′22″S, 77°51′10″W (Figure 1). Specimens were originally collected for analyses of gastrointestinal tract structure and function (German, 2009; German & Bittong, 2009), jaw morphometric analyses (Lujan & Armbruster, 2012), and dietary stable isotope analyses (German, 2009; Lujan et al., 2011). Voucher specimens of all fish species collected during this fieldwork are cataloged at the Natural History Museum of San Marcos University in Lima, Peru, and at the Auburn University Museum Fish Collection in Auburn, Alabama, USA. Fishes for this study were ethically collected, euthanized, and preserved following IACUC protocol D995 (D.P. German) at the University of Florida.

Individuals of each species were euthanized in buffered river water containing 1 g/L tricaine methanesulfonate (MS‐222; Argent Chemicals Laboratory, Inc.) before being measured (standard length) and dissected following the procedures of German and Bittong (2009). Guts from each individual fish were removed, placed in sterile Whirlpak^®^ bags, and immediately frozen, whole, in liquid nitrogen. Gut samples were transported to the USA on dry ice and kept at −80°C until used for this study. Samples were removed from the freezer, allowed to partially thaw, and were uncoiled on a chilled (~4°C), sterilized cutting board. Following German and Bittong (2009), the intestines of each fish were divided into three sections of equal length representing the proximal, mid, and distal regions. Each individual intestinal region sample was placed in its own sterile 0.5‐ml centrifuge vial, re‐frozen on dry ice, and shipped on dry ice to University of Mississippi for DNA extraction and sequencing.

Samples (*n* = 4) of the submerged coarse woody debris on which the fishes were found were also collected by scraping to a depth of ~1 cm with a razorblade and were immediately frozen, processed, transported, and stored as for gut samples.

### 2.2 DNA extraction, 16S rRNA gene sequencing, analysis

DNA was extracted from the contents of a 5‐cm section of each frozen gut sample using a MoBio PowerSoil DNA extraction kit (MoBio). After an initial step in which the gut section was thawed (37°C) and vortexed for 2 min in 200 µl of the first lysis buffer to disperse gut contents, DNA was extracted following the manufacturer's specifications. Frozen woody debris samples were scrubbed with sterile toothbrushes into the first lysis buffer solution and DNA extracted from that suspension. DNA from all extractions was amplified using a dual index barcoding approach that targets the V4 regions of the 16S rRNA gene (Jackson, Stone, & Tyler, 2015; Kozich, Westcott, Baxter, Highlander, & Schloss, 2013). Amplification products were standardized using SequalPrep Normalization plates (Life Technologies) and pooled prior to sequencing using an Illumina MiSeq platform at the Molecular and Genomics Core Facility at the University of Mississippi Medical Center.

### 2.3 Data analysis

Analysis of 16S rRNA gene sequence data was conducted in mothur (Schloss et al., 2009) v.1.40.5. Raw data files (FASTQ) were processed following the procedures recommended by Schloss, Gevers, and Westcott (2011) and Kozich et al. (2013), as updated in the online MiSeq SOP (https://www.mothur.org/wiki/MiSeq_SOP; accessed July–December 2019). Sequences were aligned to the SILVA 16S rRNA database release 132 and sequences that did not align with the V4 region or contained homopolymers >8 bp were discarded. Aligned sequences were classified to the Ribosomal Database Project release version 16 database, and any sequences that classified as nonbacterial were removed. Valid bacterial sequences were clustered into operational taxonomic units (OTUs) based on 97% sequence similarity and used for subsequent analyses of alpha and beta diversity. OTUs represented by just a single sequence in the dataset were excluded from diversity analyses, and those analyses occurred following subsampling (1,000 iterations) to a standardized number of 10,000 sequence reads per sample. Linear mixed models were conducted on the alpha diversity indices, treating the gut region as the fixed effect, with significant differences determined using Tukey's HSD test. The beta diversity index (Bray–Curtis dissimilarity) was exported from mothur into R version 3.6.1 (R Studio Team, 2019) with nonmetric multidimensional scaling (NMDS) ordinations created using the metaMDS() function in the vegan package (Oksanen et al., 2019). A permutational MANOVA was performed on the beta diversity indices, utilizing the vegdist() and adonis() functions in the vegan package. Hierarchical cluster analysis was conducted using the hclust() function from the stats package, and a cladogram was generated with the package ggplot2 (Wickham, 2016). Core bacteria were defined as OTUs present at >0.01% relative abundance in 95% of samples and were analyzed using the core_members() function of the R microbiome package (Lahti, 2017). Community networks were created based on significant (*p* < .05 and Spearman's correlation >0.7) relationships between taxa and exported into Cytoscape version 3.7.2.

### 2.4 Functional inference for wood utilization

The metagenome inference tool Piphillin (Iwai et al., 2016) was used to predict the ability of the bacterial community to metabolize lignin, cellulose, and hemicellulose. A nearest‐neighbor algorithm matched the relative abundance of our 16S rRNA gene sequences to the Kyoto Encyclopedia of Genes and Genomes (KEGG) microbial genetic database (updated October 2018) with a 97% identity cutoff to infer the metagenomic content of the bacteria (Iwai et al., 2016). Ninety‐eight orthologs that are likely involved in the processing of lignin, cellulose, and hemicellulose (Kumar et al., 2018; Santos, Sarmento, de Miranda, Henrique‐Silva, & Logares, 2019; Scully et al., 2013; Zheng et al., 2019) were selected for further analysis. MANOVAs were conducted on the relative abundance of the KEGG pathways, and representative orthologs present between catfish species, gut regions, and submerged wood, with an FDR correction of <0.05.

## 3 RESULTS

Across all samples, a total of 940,950 valid 16S rRNA gene sequences were obtained after removal of sequences that were potentially chimeric or of nonbacterial origin, for a mean of >24,000 sequence reads per sample. One sample (the distal gut of a *Pqs nocturnus* specimen) yielded low sequence counts (<1,000) despite multiple attempts and was removed from the dataset. Thus, the final dataset consisted of 940,168 sequences, which was represented by 56,655 unique sequence types, with a mean of 24,106 sequences per sample (range 12,120–59,879).

**Figure 2:**
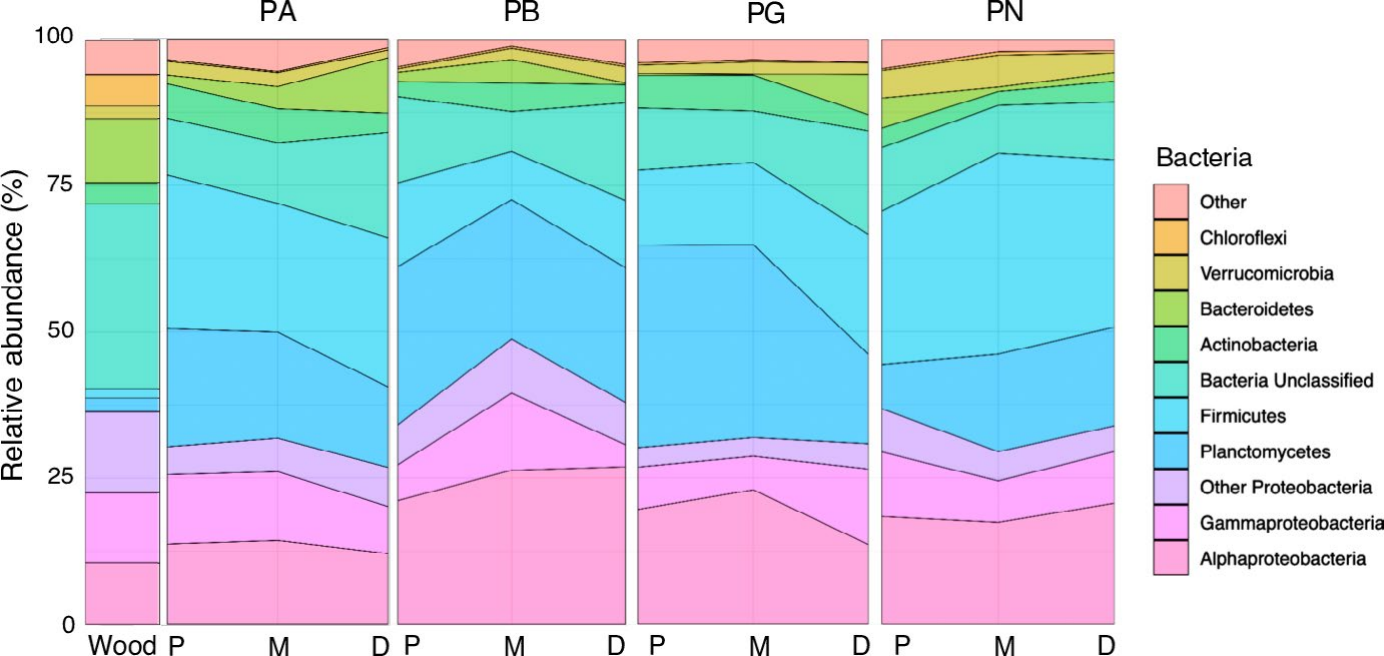
Relative abundance of dominant bacterial phyla in the gastrointestinal tracts of four co‐existing species of wood‐eating catfish (PA = *Panaqolus albomaculatus,* PB = *Panaque bathyphilus,* PG = *Panaqolus gnomus,* PN = *Panaqolus nocturnus*; *n* = 3 for each species) collected from the Marañon River, Peru. Guts were separated into proximal (P), mid (M), and distal (D) regions, and the bacterial phyla associated with samples of partially submerged wood (*n* = 4) collected from the same site are shown for comparison. Bacterial phyla were identified through next‐generation sequencing of 16S rRNA genes with a mean of 24,106 sequence reads per sample. Proteobacteria were largely dominated by subphyla Alphaproteobacteria and Gammaproteobacteria which are shown separately

Three bacterial phyla accounted for 50.7% of the sequence reads: Proteobacteria (26.9%), Firmicutes (12.4%), and Planctomycetes (11.4%), and 28.68% of sequences were unclassified at the phylum level (Figure 2). The relative abundances of Firmicutes and Planctomycetes in gut samples (18.8% and 16.6%, respectively) were significantly greater than on wood (2.9% and 4.6%, respectively), whereas unclassified bacteria were significantly more abundant on the wood (36.7%) than in the gut (16.8%) (MANOVA, *p* < .001 for all). Within Proteobacteria, the majority of sequences belonged to the Alphaproteobacteria (38.5%) and Gammaproteobacteria (29.4%), with Deltaproteobacteria (14.8%), Betaproteobacteria (6.7%), Epsilonproteobacteria (0.1%), and unclassified Proteobacteria (10.5%) constituting the remaining sequences.

Sequences grouped into 29,076 OTUs, with 8,312 of those OTUs being singletons represented by just one sequence read. These singletons were removed from the dataset, leaving 20,764 OTUs for further analyses. Twelve OTUs represented 20.1% of the total reads; each contained >10,000 sequences and accounted for 1%–3% of all sequence reads. There was no significant difference in the relative abundance of these 12 OTUs between the proximal‐, mid‐, and distal gut regions of any catfish, though there were significant differences in the relative abundance of six of these dominant OTUs between catfishes and wood (Table 1). The most abundant OTU was classified as a member of the family Hyphomicrobiaceae (Alphaproteobacteria, Rhizobiales) representing 3.0% of all sequences obtained. This OTU, along with another in the order Planctomycetales (1.1% of reads), was significantly more abundant in *Pqs. gnomus* tissues than in either *Pqs. albomaculatus* or on wood (*p* < .05, for all; Table 1). The second most abundant OTU classified as the genus *Acinetobacter* (Gammaproteobacteria, 2.2% of reads) and was similar in abundance across wood and catfish tissues, while a third OTU identified as a member of the Planctomycetaceae (Planctomycetes, 2.1% of reads) was more abundant in *Pqe. bathyphilus* tissues than in *Pqs. albomaculatus*, *Pqs. nocturnus* or on wood (*p* < .05, for all). Of the remaining dominant OTUs, two members of the order Rhizobiales (Proteobacteria, each 1.4% of reads) were significantly more abundant in *Pqe. bathyphilus* and *Pqs. nocturnus* while an OTU classified as being within the class Clostridiales (1.3% of reads) was more abundant in *Pqs. nocturnus* than other samples (*p* < .05; Table 1). Of the 20,764 OTUs, 54% (11,245) were detected in both fishes and wood samples, while 4,421 were found only in wood samples, and 5.098 were found only in fishes.

**Table 1:** Relative abundance (%) of the 12 most abundant bacterial OTUs in the guts of four wood‐eating catfish species, *Panaqolus albomaculatus* (PA)*, Panaque bathyphilus* (PB), *Panaqolus gnomus* (PG)*,* and *Panaqolus nocturnus* (PN); *n* = 3 for each species) collected from the Marañon River, Peru, as well as those associated with partially submerged wood (*n* = 4) collected at the same site

OTU Taxonomy (Phylum, Order, Genus)	Wood	PA	PB	PG	PN
Proteobacteria, Rhizobiales, Hyphomicrobiaceae unclassified	0.078^¶^	0.799^¶^	4.288	6.161^‡∆^	2.248
Proteobacteria, Pseudomonadales, *Acinetobacter*	4.186	2.971	0.333	2.333	2.330
Planctomycetes, Planctomycetales, Planctomycetaceae unclassified	0.029^§^	1.142^§^	3.71^†‡∆^	2.772	0.940^§^
Unclassified Bacteria	0.002	0.150	3.332	1.470	1.502
Bacteroidetes, Flavobacteriales, *Cloacibacterium*	0.095	3.430	1.435	1.986	1.757
Planctomycetes, Planctomycetales, Planctomycetaceae unclassified	0.067	0.920	2.601	3.354	0.454
Proteobacteria, Rhizobiales, *Methylocystis*	0.191^§∆^	0.278^§∆^	2.465^†‡¶∆^	0.927^§^	1.828^†‡§^
Proteobacteria, Rhizobiales, Rhizobiales unclassified	0.092^§^	0.852^§^	2.903^†‡¶∆^	0.738^§^	1.201^§^
Firmicutes, Clostridiales, *Hydrogenispora*	0.029	4.790	0.273	0.281	2.696
Firmicutes, Clostridiales, Clostridiales unclassified	0.001^∆^	0.028^∆^	0.001^∆^	0.001^∆^	8.913^†‡§¶^
Proteobacteria, Rhizobiales, *Bradyrhizobium*	0.138	0.819	1.661	0.957	0.973
Planctomycetes, Planctomycetales, Planctomycetaceae unclassified	0.032^¶^	0.092^¶^	0.756	4.015^†‡^	0.629

Note

Bacteria are classified to the finest taxonomic level that was resolved. ANOVAs were conducted with significant (*p* < .05) results followed by Tukey's honest significance test. Values that are significantly different to wood samples are indicated with †, while values that are significantly different to the catfish species PA, PB, PG, and PN are indicated by ‡, §, ¶, and ∆, respectively.

**Figure 3:**
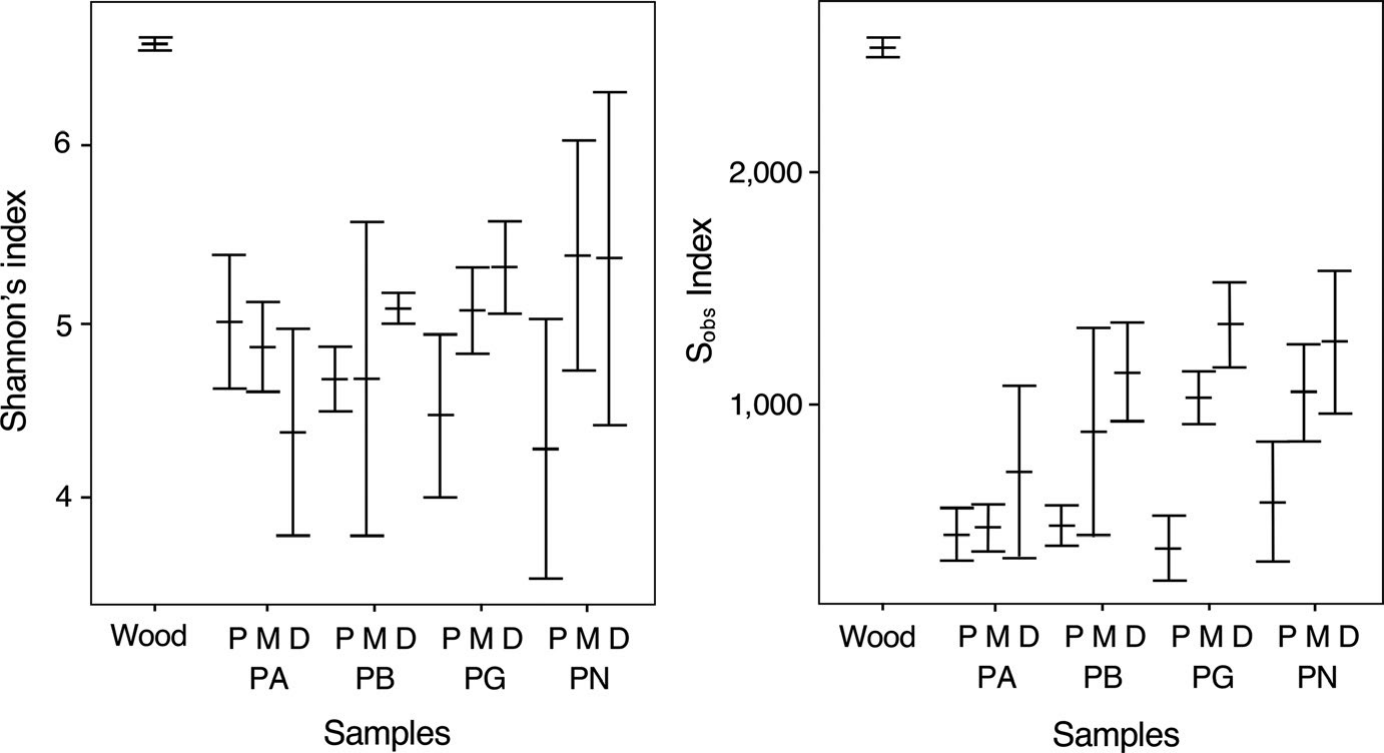
Bacterial diversity in the gastrointestinal tracts of four co‐existing species of wood‐eating catfish (PA = *Panaqolus albomaculatus,* PB = *Panaque bathyphilus,* PG = *Panaqolus gnomus,* PN = *Panaqolus nocturnus*) collected from the Marañon River, Peru, and for partially submerged wood collected from the same site. Gut samples were separated into proximal (P), mid (M), and distal (D) regions, and diversity assessed as Shannon's index (a) and observed species richness (S_obs_, b) based on repeated (1,000 times) subsampling of 10,000 random sequence reads to account for differences in sequencing depth between samples. Values are means + SE based on *n* = 3 for fish samples and *n* = 4 for wood

Following subsampling to a standardized 10,000 reads per sample, the mean coverage of the catfish gut samples was 0.98, whereas the coverage for the wood samples was lower at 0.91. The bacterial community present on the wood was significantly more diverse, based on both Shannon's index and observed species richness (S_obs_), than any region of gut tissue (*p* < .01; Figure 3). Within *Pqs. gnomus*, both the mid‐ and distal gut regions harbored a significantly richer bacterial community than the proximal gut (*p* < .05, S_obs_ index), while for *Pqe. bathyphilus* the distal gut community was significantly richer than the proximal (*p* < .05, S_obs_ index; Figure 3). There were no significant differences in bacterial microbiome richness based on gut location for either *Pqs. albomaculatus* or *Pqs. nocturnus* (*p* > .05, S_obs_ index). Further, there were no significant differences in Shannon's index of diversity between species, or between gut regions for any species (Figure 3).

**Figure 4:**
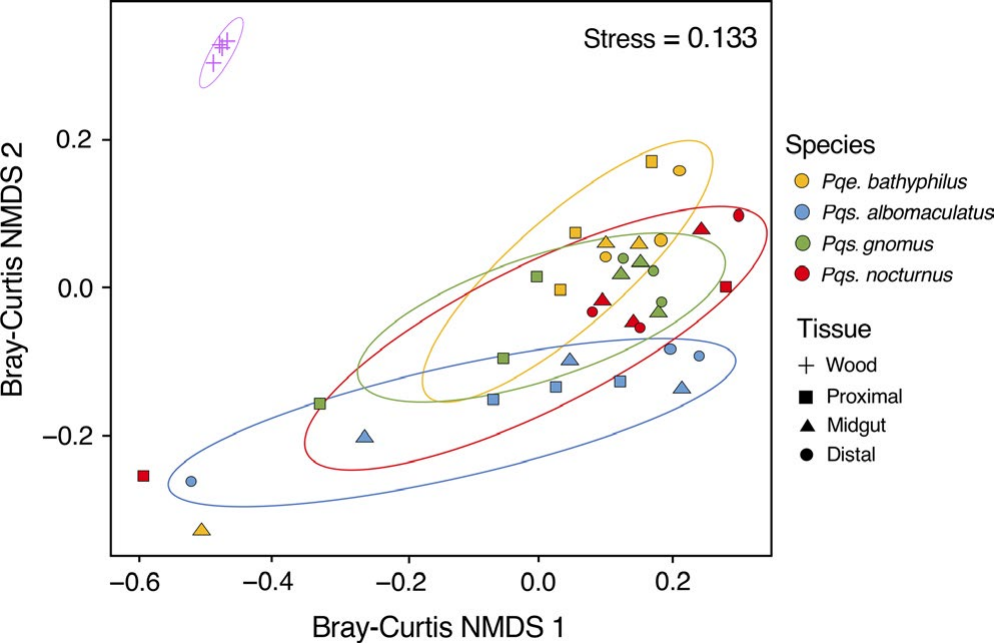
Community similarity of the gut microbiomes of four co‐existing species of wood‐eating catfish (*Panaqolus albomaculatus, Panaque bathyphilus, Panaqolus gnomus, and Panaqolus nocturnus*) collected from the Marañon River, Peru, and for partially submerged wood collected from the same site. Similarity is presented as a nonmetric multidimensional scaling (NMDS) ordination based on the Bray–Curtis dissimilarity index, with 95% confidence ellipses. Gut samples were separated into proximal, mid, and distal regions for each fish species. Permutational MANOVAs were performed to compare between the different catfish species and their gut regions

Based on the Bray–Curtis dissimilarity index, gut microbiomes of wood‐eating catfishes were significantly different from bacterial assemblages on submerged wood (AMOVA *p* < .05; Figure 4). Gut microbiomes also differed between catfish species (MANOVA *p* < .05), and there were no significant differences between any gut regions within catfish species. *Pqs. albomaculatus* and *Pqe. bathyphilus* shared a similar number, but not identity, of core OTUs, comprising approximately 6% of their entire bacterial sequences (Appendix S1). The core microbiome of *Pqs. gnomus* was expanded when compared to any other species, contributing to 15.4% of its microbiome, while *Pqs. nocturnus* had the smallest core microbiome with only 1.7% of its entire sequences found in 95% of samples. Wood had the greatest diversity within its core microbiome, with an average of 50.9% of its sequences classified as core taxa. There was significant overlap between the core bacteria and those bacteria identified through the network analysis as being important to specific catfish species (Appendix S1). Network analysis identified community differences between potentially important bacteria within catfish species, with a lack of core Firmicutes in *Pqe. bathyphilus,* fewer core Proteobacteria in *Pqs. nocturnus,* and a greater diversity of core Actinobacteria in *Pqs. gnomus* (Appendix S1). Of the four catfish species, *Pqe. bathyphilus* was more distinct in the identity of its core bacteria, which was further supported by hierarchical clustering. Wood had many core taxa identified through network analysis that were not identified within any of the fish species (Appendix S1).

**Figure 5:**
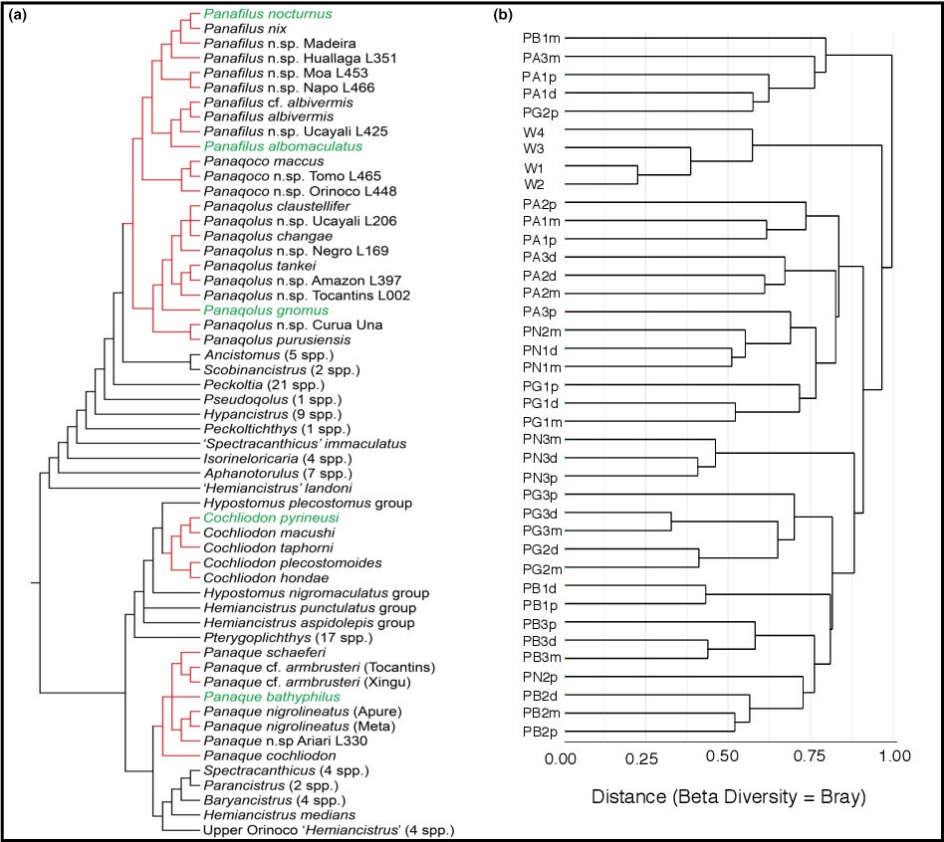
Relationships between gut microbiome composition of four wood‐eating catfish species and host phylogeny. Catfish phylogeny (a) is based on that of Lujan et al. (2017) while microbiome samples are clustered based on Bray–Curtis dissimilarity. Individual gut samples (numbered) were separated into proximal (P), mid (M), and distal (D) regions and come from three individuals of each of *Panaqolus albomaculatus* (PA)*, Panaque bathyphilus* (PB)*, Panaqolus gnomus* (PG)*,* and *Panaqolus nocturnus* (PN). Similarities to bacterial assemblages on partially submerged wood (W) collected from the same site are also shown

Hierarchical clustering of samples based on the Bray–Curtis dissimilarity index of their bacterial assemblage resulted in significant groupings of samples (PERMANOVA *p* < .001, Figure 5). Significant (*p* < .05) specific clusters identified included a cluster of wood samples and clusters of multiple individuals of each catfish species (Figure 5). Samples taken from different individuals of the species *Pqs. albomaculatus* and *Pqe. bathyphilus* clustered closely together, whereas samples taken from different individuals of *Pqs. nocturnus* and *Pqs. gnomus* clustered less tightly (Figure 5). For all species, samples taken from different gut regions of an individual were more likely to cluster together. Clustering of some gut microbiome samples reflected phylogenetic patterns in wood‐eating catfish species (e.g., *Pqe. bathyphilus* samples clustered separately from most other samples, and *Pqs. albomaculatus* grouped with some *Pqs. nocturnus* samples) but there were exceptions (e.g., some *Pqs. gnomus* samples grouped with *Pqs. bathyphilus*; Figure 5).

The KEGG Global and overview maps contained 39.3% of all proteins predicted with Piphillin (Appendix S2). Abundant pathway maps included carbohydrate metabolism (mean 8.3%), amino acid metabolism (5.6%), energy metabolism (4.8%), and xenobiotics biodegradation and metabolism (mean 4.2%; Appendix S2). There was a significantly greater abundance of three pathway maps on wood than within the wood‐eating catfish gut: glycan biosynthesis and metabolism, folding, sorting and degradation of proteins, and cell motility. There was a significantly lower abundance of proteins relating to signaling molecules and interactions on wood than within the catfish gut (Appendix S2). Relative abundance of pathways within the gut samples varied, for example, there were significantly fewer proteins related to nucleotide metabolism in each gut region of *Pqe. bathyphilus* than identified on wood, whereas for both *Pqs. albomaculatus* and *Pqs. nocturnus* only the midgut contained significantly fewer proteins (Appendix S2). Of the 25 KEGG metabolic pathway maps, 15 contained at least one significant difference between the relative abundance of proteins predicted in the catfish gut bacterial communities and those on submerged wood (Appendix S2).

Out of 97 KEGG orthologs associated with the digestion of cellulose, hemicellulose, and lignin (i.e., wood degradation), the abundance counts for 20 genes were below the cutoff of 97% identity, indicating very low counts and so were excluded from the analysis. Of the remainder, 42 orthologs differed significantly in their relative abundance between catfishes and on wood (Appendix S3). For two‐thirds (28) of these orthologs, the predicted abundance was significantly lower (*p* < .05) in the gut microbiomes of all the wood‐eating catfishes compared to the bacterial assemblage on wood (Appendix S3). Just five orthologs (K00104 glycolate oxidase, K02844 UPD‐glucose: (heptosyl) LPS α‐1,3‐glucosyltransferase, K03386 peroxiredoxin, K00694 cellulose synthase, and K01207 β‐N‐acetylhexosaminidase) were more abundant in all catfish species compared to the wood samples, and a further eight orthologs were significantly higher in some catfish species compared to wood, but not all. One ortholog was lower in two of the catfish species relative to the wood.

## 4 DISCUSSION

Our results indicate that host identity, and perhaps phylogenetic history, plays important roles in determining the gut microbiome of wood‐eating catfishes in the Amazon basin of South America, not supporting our hypothesis of microbiome similarity among co‐occurring fishes. The gut bacterial communities of different individuals in the study generally clustered according to their taxonomic identity, and each of the four species in this study had a gut microbiome that differed from that of all other species, with a distinct core microbiome. Moreover, the bacterial community of each catfish species was different from that of the wood on which they were feeding, whereas bacterial samples from different gut regions and different pieces of wood showed few to no differences. This, in congruence with previous work on captive wood‐eating catfishes (Di Maiuta et al., 2013; McDonald et al., 2012; Nelson et al., 1999; Watts et al., 2013), supports our hypothesis that the guts of these fishes harbor gut microbial communities that are distinct from those of the wood on which they graze.

The more intriguing and novel result of our study is that the gut microbiome segregated more with host identity than by any other measure—despite the apparent similarity in diet, gut morphology, and function among the studied species (German, 2009; German & Bittong, 2009). Therefore, there may be some aspect to host genetics and gut metabolism that selects for aspects of microbial community assembly. There may also be vertical transmission from parent to offspring as these fishes generally spawn in blind caves and larval fish develop under conditions in which they encounter and likely consume fecal material from their parents (Lujan et al., 2017). We observed weaker evidence of phylosymbiosis, correlation of microbiome similarity and host phylogenetic distance, but saw some indication that this phenomenon may exist at the species level. Not only did the microbiome of *Pqe. bathyphilus* cluster more tightly than the three *Panaqolus* species present, but it was also the most dissimilar to any other species, reflecting the phylogenetic distance between these host genera and species. Although this suggests a relationship between the similarity of gut microbial communities and the evolutionary distance between their hosts’, this needs to be studied further and in a broader phylogenetic context.

Gut samples from the four wood‐eating catfishes were generally dominated by different bacterial sequences affiliated with the Firmicutes, Planctomycetes, and Proteobacteria, each phylum typically accounting for at least 10% of the 16S rRNA gene sequences recovered. All three of these phyla have been identified as accounting for a substantial proportion of fish gut microbiomes in general (Clements et al., 2014; Colston & Jackson, 2016; Egerton, Culloty, Whooley, Stanton, & Ross, 2018; Ghanbari, Kneifel, & Domig, 2015; Liu et al., 2016; Moran et al., 2005, 2019; Sullam et al., 2012). Potential contributions to host function for bacteria within these phyla include roles in nutrient absorption, digestion, and immune development (Egerton et al., 2018; Ikeda‐Ohtsubo et al., 2018; Yukgehnaish et al., 2020). Similar phyla were identified within the gastrointestinal tissues of *Panaque nigrolineatus* sampled after at least three weeks in aquaria, although unlike our study their abundance varied significantly across different gut regions (McDonald et al., 2012). Further, Fusobacteria was the dominant bacterial phyla (~60%) in feces isolated from various *Panaque* species acquired from the aquarium trade (Di Maiuta et al., 2013), compared with an abundance of less than 0.1% in our gut tissues. Of the prominent OTUs that were identified, six were members of the Rhizobiales, Pseudomonadales, and Flavobacteriales, orders of Bacteria that are often considered as “plant‐growth promoting microbes” (Backer et al., 2018) but have been reported within the gut microbiota of a wide range of fish species (Estruch et al., 2015; Sullam et al., 2012). While the presence of these bacteria on decomposing wood may be related to a biocontrol function (Bloemberg & Lugtenberg, 2001) or inhibition of phytopathogens (Rani, Arundhathi, & Reddy, 2012), their increased abundance within the gut microbiota could also relate to a role in nitrogen fixation (Egamberdieva, Berg, Lindström, & Räsänen, 2010), although Lujan et al. (2011) suggest that N‐fixation is likely not important in the biology of these fishes.

A prior study on *Panaque nigrolineatus* kept in aquaria found that the proximal gut shared many phylotypes with that of the water from its aquarium, although overall the microbial communities were distinct (McDonald et al., 2012). In our study, the proportions of the Firmicutes and Planctomycetes in the gut community were substantially higher than those on the submerged wood on which these fishes were feeding, although 35 of the 47 families of Firmicutes found in fishes were also found on wood, and all four families of Planctomycetes were found in both gut and wood samples. The wood‐associated microbiome contained significantly more bacterial phyla within the Chloroflexi, as well as more that were unclassified. Just over half of all OTUs were detected in both fishes and wood, although each sample type also had its own distinct OTUs and the proportions of even the shared OTUs varied between gut and wood samples. Thus, while there were some shared members of the bacterial community, the marked difference between the wood and gut samples indicates that the gut microbiome of wood‐eating catfishes is not simply a direct reflection of bacteria that are ingested. Rather, there must be some selection for specific bacterial taxa that are capable of growth within the gut environment.

The wood‐associated bacterial community was also much more diverse; wood samples averaged almost 2,000 bacterial OTUs, compared to 300–1,000 OTUs in fish gut samples. Even the proximal gut samples had lower alpha diversity than the submerged wood, although the proximal gut should presumably contain more similar bacteria to the wood in the diet if there is no selection for specific gut bacteria. Indeed, if there was any trend within the gut regions in terms of alpha diversity, it was that the richness of OTUs increased from the proximal to mid to distal gut, a pattern seen in *Pqs. gnomus* and *Pqe. bathyphilus*. This would be expected based on microbiome studies of other vertebrates, where residual bacterial populations are passed from earlier in the digestive system to later segments (Clements et al., 2014; Moran et al., 2005), or if some of the ingested microbes themselves are digested in the proximal intestine (German & Bittong, 2009). However, the disparity in bacterial diversity between the submerged wood and the contents of the proximal gut, which presumably contained the least digested wood material, reinforces the finding that the gut microbiome of these catfish is not simply a reflection of their diet.

Consistent with previous research on multiple levels of digestive physiology (German, 2009; German & Bittong, 2009; German & Miles, 2010), there is little evidence of either microbial digestion of wood in the fishes’ guts, or of fiber degradation pathways that change along the digestive tract. We observed significant differences between KEGG pathway maps found on wood and in the catfishes guts, with most of the fiber degradation pathways involving hydrolysis of cellulose, mannose, and xylose, or their breakdown products, being equivalent, or more abundant in wood samples than in the fishes’ guts. Indeed, a quarter of all fiber degradation pathways were absent from the gut samples. German and Bittong (2009) argued that the low cellulolytic activity observed in the intestines of wood‐eating catfishes showed that these enzymes were likely ingested with their woody detrital diet and the microbiome dataset here supports that conclusion. More broadly, German and Bittong (2009), German (2009), and Lujan et al. (2011) have argued that Amazonian wood‐eating catfishes are detritivores that graze on a substrate (wood) that is common in Amazonian waters, but that these fishes are more likely reliant on fiber digestion performed by microorganisms in the environment than on fiber degradation in their guts. Thus, it seems likely that microbial residents of the catfish intestine that are involved in host digestion play roles other than fiber degradation (Moran et al., 2019). This stands in contrast to those fish taxa that are reliant on their gut microbiome to digest ingested plant material (e.g., Clements, German, Piché, Tribollet, & Choat, 2017; Moran et al., 2005; Mountfort, Campbell, & Clements, 2002).

Phylogenetic and ecological studies by others and ourselves indicate that each of the three wood‐eating catfish genera evolved independently from nonwood‐eating ancestors (Figure 5a; Lujan et al., 2017) and that co‐occurring species partition wood resources such that competition might be reduced (Lujan et al., 2011). Despite being regarded as wood‐eating catfishes, there is little evidence that the fishes digest significant amounts of wood cellulose and hemicellulose in their digestive tracts (German, 2009; German & Bittong, 2009; German & Miles, 2010), but they do appear to be equipped to digest microbial decomposers on ingested wood with their own endogenous digestive machinery, and also with the aid of enteric symbionts (German, 2009; German & Bittong, 2009). In support of this, one of the carbohydrate pathways that were more represented in the fish guts was that for β‐N‐acetylhexosaminidase, which, like N‐acetyl‐β‐d‐glucosaminidase, is part of the chitin degradation pathway, suggesting a capability of digesting the cell walls of fungi, the primary decomposers of wood in freshwater systems (Gönczöl & Révay, 1997; Maltby, 1994; Révay & Gönczöl, 1990). The only study to attempt to characterize fungi within the guts of wood‐eating catfishes was limited to two individuals of *Panaque nigrolineatus* fed different diets in an aquarium setting (Marden, McDonald, Schreier, & Watts, 2017) and while it shows the presence of a fungal gut community, it provides little insight into the potential role for these fungi or whether they reflect fungi present on the food consumed.

Every aspect of the wood‐eating catfishes’ digestive strategy points against these fishes being reliant on digestion of wood cellulose by gut microbes (German, 2009; German & Bittong, 2009; German & Miles, 2010). Four key lines of evidence support this contention. First, wood‐eating catfishes pass wood through their guts in less than four hours (German, 2009), which is incredibly fast, and not observed in other animals reliant on microbial digestion in their intestines (Clements et al., 2017; German, Sung, Jhaveri, & Agnihotri, 2015). Second, animals that digest cellulose with the aid of their gut microbiome tend to have anaerobic conditions in the gut, and elevated levels of short‐chain fatty acids (SCFAs), the byproducts of microbial fermentation, in the gut region with the densest microbial population (usually the hindgut; German et al., 2015; Karasov & Martínez del Rio, 2007; Stevens & Hume, 1998). SCFA concentrations in the guts of wood‐eating catfishes are low (<3 mM in any gut region) and do not spike in the hindgut (German & Bittong, 2009). Moreover, cellulose digestibility is low in the wood‐eating catfishes (German, 2009). Third, digestive enzyme activities in the wood‐eating catfish guts show low activity against cellulose, the opposite of which would be a prerequisite for digesting cellulose (German & Bittong, 2009). Instead, the wood‐eating catfishes have elevated activities of digestive enzymes to degrade soluble (e.g., starch, disaccharides, proteins) components of their diet (German & Bittong, 2009). Finally, a lab‐based stable isotopic tracer study showed little ability of loricariid catfishes to directly assimilate wood carbon (German & Miles, 2010). Thus, all available data indicate that wood‐eating catfishes are not reliant on their intestinal microbiome to digest wood in their guts, and this microbiome study supports that.

## 5 CONCLUSIONS

We investigated the microbial diversity in the digestive tracts of wood‐eating catfishes from the Amazon basin, finding that their microbiomes are more correlated with host species identity than any other factor. Although we see some support for phylosymbiosis, this contention needs to be investigated further. One of the important elements of our investigation is that we could examine microbial diversity and potential metabolic pathways represented in those communities based on a rich literature considering the digestive physiology of the host organisms. These previous investigations provide context to our findings, something that is lacking in many investigations of fish gut microbiomes (Clements et al., 2014). Although it is obvious that the wood‐eating catfishes do not digest wood in their guts with the aid of microbial symbionts, the roles that these symbionts play in digestion and metabolism more broadly (including immune function) and their contribution to host community ecology and competitive interactions should be the focus of future studies. At the very least, our study reinforces that just because an animal consumes a resource considered to be “recalcitrant”, it does not mean that microbial gut symbionts help facilitate digestion. Rather, “microbial digestion” can mean many different things, ranging from environmental microorganisms degrading and modifying resources before an animal even consumes them, to degradation of secondary metabolites in the animal's gut, to full digestion of the ingested material in the gut environment. Microbiome studies are only just beginning to reveal the myriad roles that microbes play in host biology (Moran et al., 2019).

## CONFLICT OF INTEREST

None declared.

## AUTHOR CONTRIBUTION

**Mark McCauley:** Data curation (equal); Formal analysis (equal); Visualization (equal); Writing‐original draft (equal); Writing‐review & editing (equal). **Donovan German:** Conceptualization (equal); Funding acquisition (equal); Investigation (equal); Methodology (equal); Project administration (equal); Writing‐original draft (equal); Writing‐review & editing (equal). **Nathan K Lujan:** Conceptualization (equal); Funding acquisition (equal); Investigation (equal); Methodology (equal); Visualization (equal); Writing‐original draft (equal); Writing‐review & editing (equal). **Colin R Jackson:** Conceptualization (equal); Funding acquisition (equal); Investigation (equal); Methodology (equal); Project administration (equal); Writing‐original draft (equal); Writing‐review & editing (equal).

## **AUTHOR** **CONTRIBUTIONS**

DPG, NKL, and CRJ: Designing the study based on past field sampling by DPG and NKL. CRJ: Conducting laboratory assays. CRJ and MM: Conducting bioinformatics and data analysis. MM, DPG, NKL, and CRJ: Manuscript writing.

## Supporting information

Appendix S1

Appendix S2

Appendix S3

Supplementary Material

## Data Availability

DNA sequences: NCBI SRA BioProject ID PRJNA579140.
