# Supplementary material for: Gut microbiomes of sympatric Amazonian wood‐eating catfishes (Loricariidae) reflect host identity and little role in wood digestion
Source: Ecol Evol. 2020 May 25;10(14):7117–28. doi: 10.1002/ece3.6413 (PMC7391310; doi:10.1002/ece3.6413)
Supplement: Supplementary file 1 — Appendix S1 [file ECE3-10-7117-s001.pdf]

Phylum	Family	Wood	*P. albomaculatus*	*P. bathyphilus*	*P. gnomus*	*P. nocturnus*
Planctomycetes	Planctomycetaceae	■	■	■	■	■
Bacteroidetes	Flavobacteriaceae	■	■	■	■	■
	Chitinophagaceae	■				
Proteobacteria	Hyphomicrobiaceae	■	■	■	■	■
	Rhizobiales (Unclassified)	■	■	■	■	■
	Bradyrhizobiaceae	■	■	■	■	■
	Methylocystaceae	■		■	■	■
	Moraxellaceae	■	■	■	■	
	Coxiellaceae	■	■	■	■	
	Rhodobacteraceae	■	■		■	
	Legionellaceae	■	■		■	
	Comamonadaceae	■	■			■
	Syntrophaceae	■				
	Enterobacteriaceae	■				
	Xanthomonadaceae	■				
	Rhizomicrobium	■				
	Labilitrichaceae	■				
	Burkholderiales	■				
	Roseiarcaceae	■				
	Proteobacteria (Unclassified)	■				
	Bacteriovoracaceae		■			
	Acetobacteraceae			■		
Firmicutes	Clostridiaceae	■	■		■	■
	Bacillaceae	■	■		■	■
	Heliobacteriaceae	■			■	■
	Veillonellaceae	■			■	■
	Firmicutes (Unclassified)	■	■			
	Ruminococcaceae	■				
	Lachnospiraceae	■				
	Erysipelotrichaceae	■				
	Peptococcaceae	■				
	Planococcaceae	■				
	Staphylococcaceae		■			
Actinobacteria	Gp6 (Unclassified)	■	■			
	Mycobacteriaceae	■				
	Rubrobacteraceae	■				
	Iamiaceae	■				
	Micrococcaceae	■				
	Nocardioidaceae		■			
	Intrasporangiaceae			■		
	Nocardioidaceae				■	
	Demequinaceae				■	
	Micromonosporaceae				■	
Acidobacteria	Gp1 (Unclassified)	■				
	Granulicella (Unclassified)	■				
	Gp18 (Unclassified)			■		
Verrucomicrobia	Verrucomicrobiaceae	■		■		
	Spartobacteria (Unclassified)	■				
Armatimonadetes	Gp2 (Unclassified)		■			
Bacteria (Unclassified)	Bacteria (Unclassified)	■	■	■	■	■
