# Supplementary material for: Gut microbiomes of sympatric Amazonian wood‐eating catfishes (Loricariidae) reflect host identity and little role in wood digestion
Source: Ecol Evol. 2020 May 25;10(14):7117–28. doi: 10.1002/ece3.6413 (PMC7391310; doi:10.1002/ece3.6413)
Supplement: Supplementary file 2 — Appendix S2 [file ECE3-10-7117-s002.pdf]

		P. bathyphilus			*P. albomaculatus*			*P. gnomus*			*P. nocturnus*		
**KEGG Metabolic Pathways**	**Wood**	**P**	**M**	**D**	**P**	**M**	**D**	**P**	**M**	**D**	**P**	**M**	**D**
**1.0 Global and overview maps**	**39.389**	**39.389**	**39.120**	**39.246**	**39.523**	**39.046**	**39.025**	**39.735**	**39.042**	**38.881**	**39.670**	**39.392**	**39.245**
**1.1 Carbohydrate metabolism**	**8.281**	**8.300**	**8.127**	**8.262**	**8.419**	**8.256**	**8.246**	**8.276**	**8.210**	**8.008**	**8.514**	**8.708**	**8.515**
**1.2 Energy metabolism**	**4.391**	**4.989‡**	**5.001‡**	**5.069‡**	**4.340**	**4.425**	**4.347**	**4.637**	**4.810**	**4.776**	**4.995‡**	**5.151‡**	**5.038‡**
**1.3 Lipid metabolism**	**2.322**	**2.289**	**2.238**	**2.236**	**2.376**	**2.396**	**2.298**	**2.276**	**2.279**	**2.227**	**2.272**	**2.227**	**2.202‡**
**1.4 Nucleotide metabolism**	**2.911**	**2.421‡**	**2.487‡**	**2.337‡**	**2.622**	**2.498‡**	**2.582**	**2.696**	**2.401‡**	**2.375‡**	**2.603**	**2.442‡**	**2.502**
**1.5 Amino acid metabolism**	**5.705**	**5.592**	**5.571**	**5.545‡**	**5.721**	**5.840**	**5.757**	**5.749**	**5.623**	**5.604**	**5.596**	**5.498**	**5.594**
**1.6 Metabolism of other amino acids**	**1.610**	**1.662**	**1.589**	**1.678**	**1.683**	**1.679**	**1.673**	**1.588**	**1.683**	**1.722**	**1.543**	**1.560**	**1.597**
**1.7 Glycan biosynthesis and metabolism**	**0.995†**	**0.825**	**0.837**	**0.790**	**0.849**	**0.823**	**0.838**	**0.847**	**0.787**	**0.763**	**0.871**	**0.824**	**0.797**
**1.8 Metabolism of cofactors and vitamins**	**3.987**	**3.837**	**3.907**	**3.813**	**3.870**	**3.730**	**3.782**	**3.835**	**3.798**	**3.835**	**3.869**	**3.804**	**3.802**
**1.9 Metabolism of terpenoids and polyketides**	**1.032**	**1.046**	**0.982**	**1.012**	**1.049**	**1.033**	**1.008**	**1.008**	**1.001**	**0.963**	**1.030**	**1.026**	**1.028**
**1.10 Biosynthesis of other secondary metabolites**	**1.656**	**1.704**	**1.718**	**1.699**	**1.699**	**1.655**	**1.684**	**1.646**	**1.739**	**1.752‡**	**1.651**	**1.664**	**1.736**
**1.11 Xenobiotics biodegradation and metabolism**	**3.679**	**4.424‡**	**4.098**	**4.559‡**	**4.383‡**	**4.514‡**	**4.403‡**	**4.118**	**4.537‡**	**4.650‡**	**3.944**	**3.975**	**3.986**
**1.12 Chemical structure transformation maps**	**0.013**	**0.018‡**	**0.018‡**	**0.019‡**	**0.015**	**0.017**	**0.017**	**0.016**	**0.019‡**	**0.019‡**	**0.018‡**	**0.017**	**0.020‡**
**2.1 Transcription**	**0.120**	**0.110**	**0.112**	**0.105**	**0.117**	**0.110**	**0.113**	**0.123**	**0.107**	**0.105**	**0.119**	**0.109**	**0.114**
**2.2 Translation**	**3.623**	**3.100**	**3.191**	**2.912**	**3.450**	**3.224**	**3.317**	**3.772**	**2.991**	**2.922**	**3.541**	**3.195**	**3.251**
**2.3 Folding, sorting and degradation**	**1.191†**	**1.043**	**1.069**	**1.013**	**1.074**	**1.039**	**1.039**	**1.105**	**1.018**	**0.999**	**1.093**	**1.057**	**1.057**
**2.4 Replication and repair**	**1.978**	**1.760**	**1.843**	**1.685**	**1.861**	**1.793**	**1.864**	**1.945**	**1.718**	**1.700**	**1.907**	**1.756**	**1.838**
**3.1 Membrane transport**	**3.643**	**3.836**	**3.959‡**	**3.911**	**3.963**	**4.030‡**	**4.225**	**3.634**	**4.018‡**	**4.131**	**3.690**	**4.001‡**	**3.855**
**3.2 Signal transduction**	**2.886**	**2.684**	**2.833**	**2.720**	**2.587**	**2.671‡**	**2.689**	**2.614**	**2.676‡**	**2.747**	**2.721**	**2.743**	**2.760**
**3.3 Signaling molecules and interaction**	**0.009†**	**0.020**	**0.018**	**0.021**	**0.017**	**0.015**	**0.015**	**0.014**	**0.020**	**0.021**	**0.015**	**0.017**	**0.020**
**4.1 Transport and catabolism**	**0.286**	**0.272‡**	**0.282**	**0.271‡**	**0.270‡**	**0.291**	**0.271‡**	**0.276**	**0.272‡**	**0.263‡**	**0.281**	**0.276**	**0.265‡**
**4.2 Cell growth and death**	**0.781**	**1.052‡**	**1.055‡**	**1.112‡**	**0.903**	**0.956‡**	**0.906**	**0.876**	**1.096‡**	**1.134‡**	**0.917‡**	**1.023‡**	**1.054‡**
**4.3 Cellular community - eukaryotes**	**0.000**	**0.000**	**0.000**	**0.000**	**0.000**	**0.000**	**0.000**	**0.000**	**0.000**	**0.000**	**0.000**	**0.000**	**0.000**
**4.4 Cellular community - prokaryotes**	**2.947**	**3.158**	**3.144**	**3.349**	**2.990**	**3.414**	**3.376**	**3.040**	**3.367**	**3.390**	**2.936**	**3.265**	**3.137**
**4.5 Cell motility**	**1.117†**	**0.825**	**0.953**	**0.854**	**0.879**	**0.980**	**1.028**	**0.745**	**0.994**	**1.079**	**0.798**	**0.893**	**0.972**
