# Supplementary material for: Gut microbiomes of sympatric Amazonian wood‐eating catfishes (Loricariidae) reflect host identity and little role in wood digestion
Source: Ecol Evol. 2020 May 25;10(14):7117–28. doi: 10.1002/ece3.6413 (PMC7391310; doi:10.1002/ece3.6413)
Supplement: Supplementary file 3 — Appendix S3 [file ECE3-10-7117-s003.pdf]

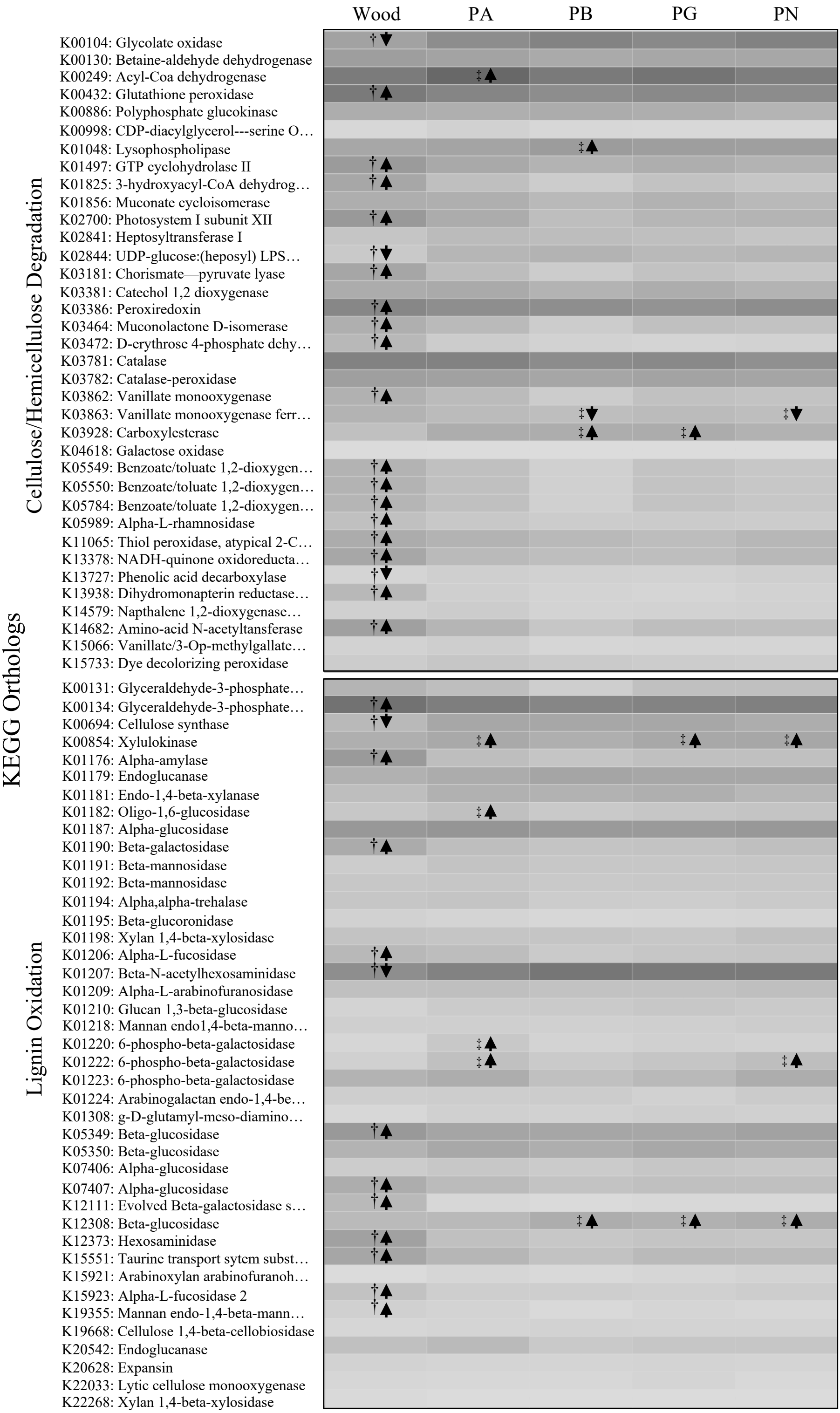
Wood
PA
PB
PG
PN
KEGG Orthologs
Cellulose/Hemicellulose Degradation
Lignin Oxidation
K00104: Glycolate oxidase
K00130: Betaine-aldehyde dehydrogenase
K00249: Acyl-Coa dehydrogenase
K00432: Glutathione peroxidase
K00886: Polyphosphate glucokinase
K00998: CDP-diacylglycerol---serine O…
K01048: Lysophospholipase
K01497: GTP cyclohydrolase II
K01825: 3-hydroxyacyl-CoA dehydrog…
K01856: Muconate cycloisomerase
K02700: Photosystem I subunit XII
K02841: Heptosyltransferase I
K02844: UDP-glucose:(heposyl) LPS…
K03181: Chorismate—pyruvate lyase
K03381: Catechol 1,2 dioxygenase
K03386: Peroxiredoxin
K03464: Muconolactone D-isomerase
K03472: D-erythrose 4-phosphate dehy…
K03781: Catalase
K03782: Catalase-peroxidase
K03862: Vanillate monooxygenase
K03863: Vanillate monooxygenase ferr…
K03928: Carboxylesterase
K04618: Galactose oxidase
K05549: Benzoate/toluate 1,2-dioxygen…
K05550: Benzoate/toluate 1,2-dioxygen…
K05784: Benzoate/toluate 1,2-dioxygen…
K05989: Alpha-L-rhamnosidase
K11065: Thiol peroxidase, atypical 2-C…
K13378: NADH-quinone oxidoreducta…
K13727: Phenolic acid decarboxylase
K13938: Dihydromonapterin reductase…
K14579: Napthalene 1,2-dioxygenase…
K14682: Amino-acid N-acetyltransferase
K15066: Vanillate/3-Op-methylgallate…
K15733: Dye decolorizing peroxidase
K00131: Glyceraldehyde-3-phosphate…
K00134: Glyceraldehyde-3-phosphate…
K00694: Cellulose synthase
K00854: Xylulokinase
K01176: Alpha-amylase
K01179: Endoglucanase
K01181: Endo-1,4-beta-xylanase
K01182: Oligo-1,6-glucosidase
K01187: Alpha-glucosidase
K01190: Beta-galactosidase
K01191: Beta-mannosidase
K01192: Beta-mannosidase
K01194: Alpha,alpha-trehalase
K01195: Beta-glucoronidase
K01198: Xylan 1,4-beta-xylosidase
K01206: Alpha-L-fucosidase
K01207: Beta-N-acetylhexosaminidase
K01209: Alpha-L-arabinofuranosidase
K01210: Glucan 1,3-beta-glucosidase
K01218: Mannan endo1,4-beta-manno…
K01220: 6-phospho-beta-galactosidase
K01222: 6-phospho-beta-galactosidase
K01223: 6-phospho-beta-galactosidase
K01224: Arabinogalactan endo-1,4-be…
K01308: g-D-glutamyl-meso-diamino…
K05349: Beta-glucosidase
K05350: Beta-glucosidase
K07406: Alpha-glucosidase
K07407: Alpha-glucosidase
K12111: Evolved Beta-galactosidase s…
K12308: Beta-glucosidase
K12373: Hexosaminidase
K15551: Taurine transport sytem subst…
K15921: Arabinoxylan arabinofuranoh…
K15923: Alpha-L-fucosidase 2
K19355: Mannan endo-1,4-beta-mann…
K19668: Cellulose 1,4-beta-cellobiosidase
K20542: Endoglucanase
K20628: Expansin
K22033: Lytic cellulose monooxygenase
K22268: Xylan 1,4-beta-xylosidase
