# Supplementary material for: Gut microbiomes of sympatric Amazonian wood‐eating catfishes (Loricariidae) reflect host identity and little role in wood digestion
Source: Ecol Evol. 2020 May 25;10(14):7117–28. doi: 10.1002/ece3.6413 (PMC7391310; doi:10.1002/ece3.6413)
Supplement: Supplementary file 4 — Supplementary Material [file ECE3-10-7117-s004.docx]

**Appendices**

**Appendix 1.** Presence/absence of bacterial families in the core microbiome, or identified as significant in the network analysis of the gut bacteria in four wood-eating catfish species, *Panaqolus albomaculatus, Panaque bathyphilus*, *Panaqolus gnomus,* and *Panaqolus nocturnus* (n=3 for each species), and on submerged wood (n=4) collected from the Marañon River, Peru. The core microbiome was categorized as bacteria present at the relative abundance of <0.01% and identified in at least 95% of the gut samples. Network analysis was conducted on significant (p<0.05) bacterial relationships with Spearman's correlation of greater than 0.7.

**Appendix 2.** Relative abundance (%) of major KEGG metabolic pathways in bacterial assemblages in the guts of four wood-eating catfish species, *Panaqolus albomaculatus, Panaque bathyphilus, Panaqolus gnomus,* and *Panaqolus nocturnus*, collected from the Marañon River, Peru, as well as those associated with partially submerged wood at the same site. Gut samples were separated into proximal (P), mid (M), and distal (D) regions for each fish species. Pathways are predicted from 16S rRNA gene sequence data using Piphillin. Wood values that are significantly different (p<0.05) to all gut samples are indicated with †, whereas gut regions that are significantly different (p<0.05) to wood are indicated by ‡. Sample size is n=3 for each catfish species and n=4 for wood.

**Appendix 3.** Patterns in the abundance of KEGG orthologs related to wood utilization (cellulose/hemicellulose degradation, lignin oxidation) in bacterial assemblages in the guts of four wood-eating catfish species, *Panaqolus albomaculatus* (PA)*, Panaque bathyphilus* (PB), *Panaqolus gnomus* (PG)*,* and *Panaqolus nocturnus* (PN), collected from the Marañon River, Peru, as well as those associated with partially submerged wood at the same site. Pathways are predicted from 16S rRNA gene sequence data using Piphillin. Darker shading indicates a greater abundance in that ortholog for that sample type relative to other samples and orthologs. Orthologs which are significantly different (p<0.05) in wood compared to all gut samples are indicated with †, whereas gut regions that are significantly different (p<0.05) to wood are indicated by ‡. Data are pooled from three individual fish x three gut regions (proximal, mid, distal) for catfish samples and from four separate samples for wood.
